# Examining the Testing Effect in University Teaching: Retrievability and Question Format Matter

**DOI:** 10.3389/fpsyg.2018.02412

**Published:** 2018-12-04

**Authors:** Sven Greving, Tobias Richter

**Affiliations:** Department of Psychology IV – Educational Psychology, Institute of Psychology, University of Würzburg, Würzburg, Germany

**Keywords:** testing effect, university teaching, retrieval practice, question format, educational psychology, net testing effect, desirable difficulties

## Abstract

Review of learned material is crucial for the learning process. One approach that promises to increase the effectiveness of reviewing during learning is to answer questions about the learning content rather than restudying the material (testing effect). This effect is well established in lab experiments. However, existing research in educational contexts has often combined testing with additional didactical measures that hampers the interpretation of testing effects. We aimed to examine the testing effect in its pure form by implementing a minimal intervention design in a university lecture (*N* = 92). The last 10 min of each lecture session were used for reviewing the lecture content by either answering short-answer questions, multiple-choice questions, or reading summarizing statements about core lecture content. Three unannounced criterial tests measured the retention of learning content at different times (1, 12, and 23 weeks after the last lecture). A positive testing effect emerged for short-answer questions that targeted information that participants could retrieve from memory. This effect was independent of the time of test. The results indicated no testing effect for multiple-choice testing. These results suggest that short-answer testing but not multiple-choice testing may benefit learning in higher education contexts.

## Introduction

Learners tend to remember less learning content when reading or listening to it only once (e.g., Aiken et al., 1975). Students often need to review the learned material, for example, when studying for exams. One potentially effective review strategy is the active retrieval of learned material from memory, which can be prompted by testing knowledge of the learned content. The finding that testing is superior to restudying the learning material is called the testing effect or retrieval practice effect (Roediger and Karpicke, 2006a). The superiority of testing compared to restudying might not be detected until later criterial tests or exams. Because of this latent effect, testing or retrieval practice is sometimes regarded as a desirable difficulty (Bjork, 1994). Desirable difficulties are defined as learning occasions that may hamper learning in the short run but enhance learning in the long run.

The testing effect is a robust finding in laboratory settings (e.g., Roediger and Karpicke, 2006b; Rowland, 2014; Karpicke, 2017), which has led researchers and practitioners to implement testing in applied educational contexts to promote the retention of learning content. Recent research has demonstrated the superiority of testing compared to restudying in various pedagogical settings (e.g., Karpicke, 2017, Table 2). Based on these findings, several authors have advocated the use of tests in educational contexts to improve learning (McDaniel et al., 2007b; Dunlosky et al., 2013; Dunn et al., 2013; Dunlosky and Rawson, 2015).

Despite the promising results and recommendations, the generalizability to educational contexts and the conditions under which the effects occur remain an open question. Based on a review of key findings from lab experiments and a discussion of studies investigating the testing effect in real-world educational settings, we argue that many of the extant field studies suffer from limitations regarding the generalizability of the results. These limitations stem mostly from methodological problems such as a third variable that cofounds the comparison of testing vs. restudying. In this article, we refer to the pure (unconfounded) difference between testing and restudying as the *net testing effect*. The aim of the present study was to examine the net testing effect in the real-world educational context of a university lecture.

### The Testing Effect in Laboratory Settings

The testing effect has been a major focus of lab-based memory research for more than a century. Summarizing this research, recent meta-analyses by Phelps (2012); Rowland (2014), and Adesope et al. (2017) found a positive average testing effect with a medium to large effects size (Cohen’s *d*/Hedges’ *g*) ranging from 0.50 to 0.61. These meta-analyses also have identified moderators of the testing effect that are potentially relevant for applications in educational contexts.

Two factors that reliably affect the testing effect are feedback (Rowland, 2014; Adesope et al., 2017) and retrievability (Rowland, 2014). The provision of feedback, mostly in the form of presenting the correct answer, seems to increase the testing effect. Retrievability in this context describes the success with which learning content can be retrieved from memory, resulting in correct responses in the testing condition. Therefore, retrievability can be operationalized by the (reverse-scored) difficulty of items in the practice tests.

Conflicting results have been reported for different question formats used in the practice tests. In the meta-analysis by Adesope et al. (2017), multiple-choice questions elicited stronger testing effects than short-answer questions, whereas Rowland (2014) reported the opposite. Furthermore, a match between question format in the testing conditions and question format in the criterial tests seems to increase the testing effect according to the meta-analysis by Adesope et al. (2017), whereas this effect was not found by Rowland. In contrast to Adesope et al. (2017), Rowland excluded applied research in his meta-analysis. Therefore, the divergent results of the two meta-analyses might reflect a moderating role of question format in educational contexts.

### The Testing Effect in Educational Contexts

The robust testing effect found in laboratory experiments has spawned a growing body of research in educational contexts. One of the first studies of this kind was a study by McDaniel et al. (2007b). In this study, college students either took weekly quizzes in the form of short-answer questions or multiple-choice questions or they restudied previously learned content. Each condition was followed by feedback. In a later criterial test, short-answer testing led to a more pronounced testing effect than did multiple-choice testing.

Since then, the testing effect has been demonstrated in different age groups (for a review, see Dunlosky et al., 2013) and with learning materials of varying complexity (for a review, see Karpicke and Aue, 2015). Three meta-analyses (Bangert-Drowns et al., 1991; Adesope et al., 2017; Schwieren et al., 2017) reported a positive testing effect in educational contexts. Bangert-Drowns et al. (1991) included only research conducted in classrooms and reported a positive testing effect with an effect size of *d* = 0.54 for studies that compared testing and no testing. Adesope et al. (2017) analyzed all studies investigating the testing effect and included study setting (classroom vs. laboratory) as a moderator. This meta-analysis estimated a positive testing effect with an effect size of *g* = 0.67 for classroom settings. Finally, Schwieren et al. (2017) reported a positive testing effect of *d* = 0.56 for studies in which psychological learning content was taught in the classroom.

Although there seems to be a consensus among researchers that the testing effect occurs in real-world educational settings, little is known about factors that moderate the effect in such settings. Several studies have validated the moderating effects of feedback found in laboratory research in applied educational contexts (McDaniel et al., 2007b; Vojdanoska et al., 2010; Marsh et al., 2012; Downs, 2015). Moreover, studies suggest that the testing effect can be found with different question formats in the practice tests (McDaniel et al., 2012; McDermott et al., 2014; Stenlund et al., 2016). The match between question formats in testing and criterial tests does not seem to matter (McDermott et al., 2014).

### Limitations of Previous Research on the Testing Effect in Educational Contexts

Numerous studies have investigated the testing effect in real-world educational contexts. However, many of these studies provide only limited information on the current research question because of internal or external validity problems that hamper the interpretation of the results.

One limiting feature of many extant studies on the testing effect in applied contexts is a lack of randomization. Because of practical constraints, researchers have often employed a quasi-experimental design, for example, by varying independent variables between courses, sections, or years (Leeming, 2002; Cranney et al., 2009; Mayer et al., 2009; Vojdanoska et al., 2010; Khanna, 2015; Batsell et al., 2017). The internal validity of these studies is questionable, because the extent that differences between the testing and the control condition are attributable to other (uncontrolled) differences between the groups is uncertain.

Other studies are limited because they lack a restudy control condition but compare the testing condition to conditions in which no exposure to information subsequent to the initial learning took place (McDaniel et al., 2007a, 2011, 2013; Johnson and Kiviniemi, 2009; Mayer et al., 2009; Vojdanoska et al., 2010; Lyle and Crawford, 2011; Roediger et al., 2011; Marsh et al., 2012; Shapiro and Gordon, 2012; Bell et al., 2015; Downs, 2015; Khanna, 2015; Batsell et al., 2017; Foss and Pirozzolo, 2017). In these studies, the testing effect is confounded with differences in exposure to and engagement with learning content, which severely limits the interpretation of their findings. To assess the magnitude of the testing effect in applied educational settings, comparing testing conditions with restudy conditions or other activities that are assumed to promote the retention of information is essential (for examples, see Adesope et al., 2017; Rummer et al., 2017).

A third limitation threatening the internal validity is found in studies that allow participants to repeat tests on the same subject. Some studies limit the amount of repetitions (Wiklund-Hörnqvist et al., 2014) while others do not (Johnson and Kiviniemi, 2009; McDaniel et al., 2012; Bell et al., 2015; Downs, 2015; Yong and Lim, 2016). Even when participants are also free to restudy the material as often as they like, it remains unclear whether differences in learning outcomes are solely attributable to testing vs. no testing or whether additional factors (e.g., differential effects of motivation) influence the number of repetitions and thus the learning outcomes.

A fourth limitation is that many studies combine the testing conditions with feedback (Leeming, 2002; McDaniel et al., 2007a,b, 2011, 2012; Carpenter et al., 2009; Cranney et al., 2009; Lyle and Crawford, 2011; Wiklund-Hörnqvist et al., 2014; Bell et al., 2015; Downs, 2015; Stenlund et al., 2017). Research has shown that testing may profit from feedback in educational settings (Vojdanoska et al., 2010). However, feedback also provides an additional study opportunity and thus an additional exposure to the learning content. We therefore argue that effects obtained in studies that combined testing with feedback cannot be readily interpreted in terms of a testing effect.

A fifth limitation is present in so-called open-label studies (Bing, 1984; Daniel and Broida, 2004; Batsell et al., 2017). In such studies, participants are told beforehand whether the learning content is tested or not, which might alter learning behavior and strategies between conditions when learning (Finley and Benjamin, 2012). As a consequence, differences obtained in testing vs. no-testing conditions can be due to differences in learning behavior that learners in the testing condition engage in, because they anticipate learning content. That is, the differences might not be due to the testing effect.

Furthermore, the internal validity is threatened in studies that feature high-stakes testing conditions (Leeming, 2002; Lyle and Crawford, 2011; Batsell et al., 2017). In these studies, participants’ scores in the testing condition affect the participants’ grades. This fact hampers the interpretation of testing effects in two ways. First, unannounced high-stakes tests have been shown to reduce the benefit of testing in applied educational settings compared to unannounced low-stakes tests (Khanna, 2015). Second, whenever open-label studies also include high-stakes testing conditions, students might alter their learning behavior and strategies, because they are motivated to get good grades.

Finally, some researchers have opted to avoid the difficulties associated with implementing experimental designs in real-world educational settings by conducting lab-based studies with “educationally relevant materials” (Butler and Roediger, 2007; Einstein et al., 2012; Marsh et al., 2012; Stenlund et al., 2016; Yong and Lim, 2016). This approach neglects the problem that the learning in secondary or postsecondary courses is likely to differ in terms of motivation, personal involvement, and effort from learning only for the purpose of participating in a psychological or educational study. These differences pose a threat to the external validity of such studies and limit their generalizability to the testing effect in actual educational settings.

### Theoretical Framework and Rationale of the Present Study

The aim of the present study was to examine the testing effect in an authentic educational setting of a university lecture with an experimental design that minimizes the issues that limit the validity of previous field studies. We used an experimental design that compared testing on a single occasion without the provision of feedback with a restudy condition. Furthermore, participants’ results in the testing conditions would not affect their grades and participants would not know the type of review condition to expect after learning.

Investigating the testing effect in this fashion is informative for a number of reasons. First, most field experiments to date include features that limit the interpretation of the results. In order to investigate the net testing effect in educational contexts, we excluded all features that might cloud the interpretability of this effect. Furthermore, in real world educational contexts, it is not always possible to provide feedback during practice tests or to provide multiple opportunities to practice retrieval. Furthermore, a single opportunity to practice retrieval without feedback makes low demands on time and personal resources compared to multiple retrieval practice opportunities with feedback. Investigating whether testing on a single occasion without feedback is effective can thus be relevant for future research and practitioners alike.

Most theories of the testing effect assume that even in this minimalistic setting, retrieval would be more beneficial for retention than restudying. The desirable difficulty framework (Bjork, 1994), the new theory of disuse (e.g., Bjork and Bjork, 2011), and the retrieval effort hypothesis (Pyc and Rawson, 2009) all incorporate the assumption that effortful retrieval should lead to better retention of that learning content and thus testing should lead to better retention than does restudying. However, it should be noted that in all of these theoretical notions retrievability plays a crucial role. Whenever the correct information cannot be retrieved from memory, no beneficial effects compared to restudying may be expected (e.g., Jang et al., 2012).

It has been repeatedly argued that multiple-choice questions and short-answer questions differ in the effort needed to be answered correctly and—given these theoretical underpinnings—should consequently lead to different testing effects (e.g., Karpicke, 2017). These different testing effects have already been demonstrated in educational contexts (McDaniel et al., 2007b).

Researchers and practitioners do not always use verbatim repetitions of retrieval practice in criterial tests and exams. Instead, questions are used that ask for related information. Previous studies suggest that these questions may lead to impaired retrieval—a phenomenon dubbed retrieval induced forgetting (for an overview, see Bjork et al., 2014)—and that this impairment depends on the question format (Carroll et al., 2007). Furthermore, research has also demonstrated that retrieval practice promoted retention of learning content not subject to retrieval practice (for an overview, see Pan and Rickard, 2018) and that the design of multiple-choice questions may affect whether unrelated learning content benefits from retrieval practice (Little et al., 2012). To investigate the potential moderating role of question format, we implemented two different testing conditions, one with short-answer questions and the other with multiple-choice questions in the practice test.

The experiment was conducted in a university lecture with minimal intervention. Therefore, the learning content was the regular course material and the lecture was held as usual. The intervention took place in the last 10 min of a 90-min lesson. Furthermore, we measured learning outcomes (i.e., memory for the learning content) in criterial tests at three different times: before and after the final exam and half a year after the final exam. In the criterial tests, we also included questions that were not targeted in the testing conditions but contained related information as well as questions that targeted learning content not subject to testing or restudy, in order to control for differential effects of these question types on multiple-choice and short-answer testing.

We expected a positive testing effect to occur. Furthermore, we examined as exploratory research questions whether the testing effect would depend on question format in the practice tests, the time of the criterial test, and retrievability. We reasoned that short-answer questions would be more suitable for prompting active retrieval of knowledge, leading to a stronger testing effect. Moreover, assuming that testing is a desirable learning difficulty, the benefits of testing vs. restudying might become visible, particularly at later criterial tests. Finally, retrievability might matter because the testing effect can only occur when retrieval is successful, especially when no feedback is given for responses in the practice tests.

## Materials and Methods

### Participants

Participants were 137 undergraduate students in their first semester, most of them female (71%) and students of psychology (92%). They participated in at least one lecture session and one criterial test. All students gave their informed and written consent prior to participation. Participants’ age ranged between 18 and 74 with a mean age of 23.15 (*SD* = 7.74).

### Materials

#### Test Questions and Restudy Statements

The content of seven lecture sessions of an introductory lecture in cognitive psychology was surveyed and 24 information units per session were identified. For each information unit, one summarizing statement, one short-answer question and one multiple-choice question were created. Statements were created by summarizing the key information of the information unit in one sentence (e.g., “Prosopagnosia is a cognitive disorder of face perception in which the ability to recognize faces is impaired to the extent that the person becomes blind to faces.”). Short-answer questions were created by asking for the key information of the information unit (e.g., “What is prosopagnosia?”). Multiple-choice questions were created by adding four response options with only one correct answer to the short-answer question [e.g., “What is prosopagnosia? (A) face blindness, (B) shape blindness, (C) color blindness, (D) object blindness”].

#### Revision Materials

For each of the seven lecture sessions, eight information units were randomly drawn from the 24 information units prepared for this session. Based on the selected information units, revision materials were prepared for each lecture session. The revision materials consisted of a one-page questionnaire asking for basic demographic information and two pages of revision items corresponding to the selected information units, consisting of either (a) eight summarizing statements (restudy condition), (b) eight short-answer questions (testing, short-answer questions), or (c) eight multiple-choice questions (testing, multiple-choice condition). In all three versions, information units were presented in the same order with four information units on each page.

#### Criterial Tests

Three criterial tests (Criterial Tests 1 to 3) were constructed that consisted of questions based on the pool of 24 information units determined for each of the seven lecture sessions. The pool of questions was expanded by creating alternate versions of the questions used in the revision material. Alternate questions were created by asking for the key information in another way (e.g., “What is the medical term for face blindness?”). For each information unit, an alternate short-answer question and an alternate multiple-choice question were created.

Each of the three criterial tests consisted of three components: (a) questions corresponding to information units included in the revision materials, (b) questions corresponding to information units not included in the revision materials, and (c) alternate questions, corresponding to information units but not identical to questions included in the revision materials. Additionally, questions previously asked in criterial tests were also included in Criterial Tests 2 and 3. Table 1 depicts the composition of the criterial tests and the total number of questions per criterial test. Most notably, the composition of Criterial Test 3 differed from the composition of the other two criterial tests. This difference was due to a sampling error in the composition of the criterial tests.

**Table 1:** Criterial test composition by components and repetition of questions in later criterial tests.

	Criterial Test 1			Criterial Test 2			Criterial Test 3		
	Questions included in study material			Questions included in study material			Questions included in study material		
Previously tested in criterial test	Verbatim	Alternate	New questions	Verbatim	Alternate	New questions	Verbatim	Alternate	New questions
Yes				7	7	7	7	7	7
No	14	14	14	14	14	14	0^a^	0^a^	7
Total		42			63			28	

*^a^Not included because of a sampling error in the composition of the criterial tests.*

Each criterial test consisted of short-answer questions and multiple-choice questions in equal proportions. Two versions were created (Versions A and B) by altering the order of questions and the question format (i.e., multiple-choice questions vs. short-answer questions) of the same question between criterial test versions so that all multiple-choice questions in Version A were short-answer questions in Version B and vice versa. All study materials are made available upon request to interested researchers.

#### Scoring

Multiple-choice questions were scored with 1 when only the correct option was ticked (correct answer) vs. 0 when a distractor was ticked or no response was given (incorrect or missing response). Short-answer questions were scored with 1 (correct response) vs. 0 (incorrect or missing response). Two independent raters scored all responses to short-answer questions. Inter-rater reliability was high across all lectures and criterial tests (6855 observations, Cohen’s κ = 0.87) and thus scores from only one rater was included in the analyses. The performance scores based on both question types served as dependent variable.

### Procedure

#### General Procedure

The study was conducted over a period of two semesters. In the first semester (October 2015–February 2016), a weekly introductory psychology lecture was taught that covered basic principles of cognitive psychology. In lecture Sessions 4–10, the manipulation of review condition (testing with multiple-choice or short-answer questions) took place. The three criterial tests, which assessed the learning of content taught in the seven lecture sessions, were administered unannounced to the students at scheduled times after the last lecture with learning content (i.e., after Session 10). Criterial Test 1 was administered 1 week after Session 10. Criterial Test 2 was administered in the first session of the second semester (April 2016–July 2016), 12 weeks after Session 10, and Criterial Test 3 was administered in the final session of the second semester, 23 weeks after Session 10.

#### Procedure During the Lecture Sessions

In each of the lecture Sessions 4–10, the last 10 min were reserved for the manipulation of the review condition. Participation was voluntary. Students were allowed to leave the lecture hall after the end of the regular lecture. Research assistants then administered the review materials, assigning participants randomly to one of the three review conditions (testing with multiple-choice questions, testing with short-answer questions, or restudy). Participants first filled in basic demographic information. They were then given 4 min to complete each page of the two pages of revision items. This was the sole opportunity to review the learning content according to one of the three conditions. Finally, participants were thanked for their participation, and the materials were collected.

#### Criterial Tests

All students present in the respective lecture sessions were allowed to take Criterial Test 1, 2, or 3, irrespective of previous participation in the study. In each of these sessions, the two versions of the criterial test were then administered in an alternating way so that participants sitting next to each other received different versions. Students were allowed 45 min to complete the test and could leave when they finished.

### Design

The design was a 3 × 3 within-subjects design with the independent variables review condition (multiple-choice test, short-answer test, restudy) and time of test (Criterial Tests 1–3 at 1, 12, and 23 weeks after the final lecture session). Each participant received one of two versions of each criterial test, which differed in format (short-answer vs. multiple-choice question) and order of questions. The dependent variable was the performance (percent correct) on the multiple-choice and short-answer questions in the criterial tests.

**FIGURE 1:**
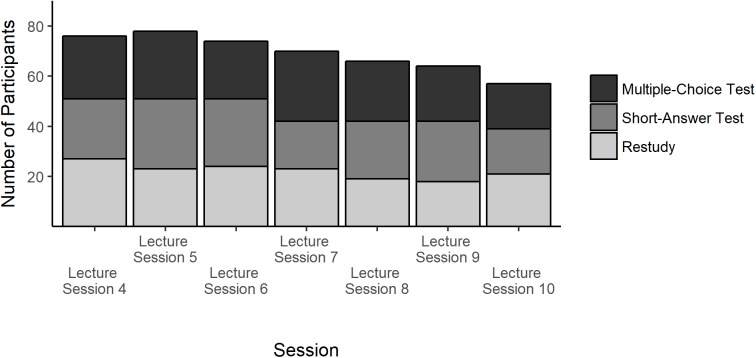
Participation by condition and session.

The design was implemented by randomly assigning participants in Sessions 4–10 of the focal lecture to one of the three review conditions. Likewise, participants were assigned to one of the two test versions of the criterial tests administered at each time of test. Figure 1 depicts the number of participants that were assigned to each review condition in the seven lecture sessions. The random allocation led to equal distributions of participants across review conditions. Similarly, participants were evenly distributed to the criterial test versions (Versions A:B) in Criterial Tests 1 (*n* = 32:33), 2 (*n* = 40:40), and 3 (*n* = 25:28). We assume that missing data is missing completely at random and thus inferences can proceed by analyzing the observed data only (Ibrahim and Molenberghs, 2009).

## Results

We estimated generalized linear mixed effect models (GLMMs) with a logit-link function (Dixon, 2008) with the R package lme4 (Bates et al., 2015).

For comparisons between conditions and extracting mean performance scores for different experimental conditions the R package lsmeans was used (Lenth, 2016). For all significance tests, Type I error probability was set to 0.05 (one-tailed for testing directed hypotheses). Participants and test items were included as random effects (random intercepts) in all models.

Separate models were estimated to examine the testing effect based on short-answer questions and the testing effect based on multiple-choice questions. In each of the two models, the testing condition was compared to the restudy condition that involved reading the summarizing statements that provided the correct answer (dummy-coded: testing = 1, restudy = 0). We additionally tested whether the testing effect depended on the time of the criterial test by including two dummy-coded predictors for Criterial Test 2 and Criterial Test 3 (Criterial Test 1 was the reference condition coded with 0 in both predictors) and the interactions of these predictors with testing vs. restudying. In addition, the models included the retrievability of learned information in form of two dummy-coded predictors that contrasted items of medium retrievability and low retrievability with items of high retrievability as the reference condition. We examined whether higher retrievability rates were associated with a larger testing effect. To construct this predictor, we grouped the short-answer questions and the multiple-choice questions separately into three equally sized, ordered categories (tertiles) according to their difficulty in the practice tests. To avoid distortions from extreme values, we discarded the lowest and the highest 5% of the distribution before the grouping. Item difficulties to the multiple-choice questions were corrected for guessing. For each of the two item types (short-answer and multiple-choice questions), grouping resulted in three categories of items with high (short-answer questions: item difficulties from 46 to 81%; multiple-choice questions: 78–100%), medium (short answer questions: 25–45%; multiple-choice questions: 53–77%), or low retrievability (short answer questions: 5–24%; multiple-choice questions: 0–53%). Finally, the models included the interaction of retrievability with testing vs. restudying. All predictors and their interactions were entered simultaneously in the models.

### Effects of Testing With Short-Answer Questions

**FIGURE 2:**
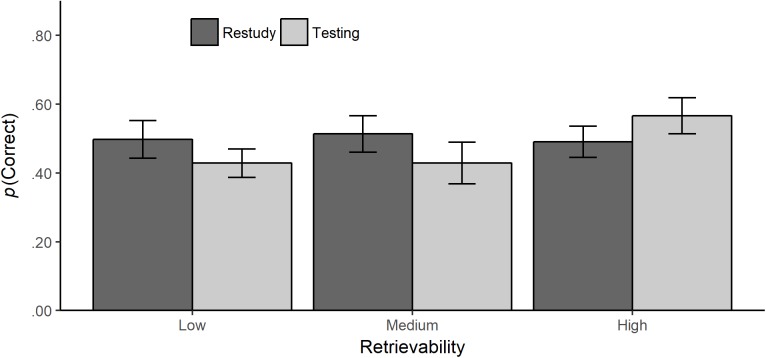
Testing with short-answer questions: mean probability of correct responses (with standard errors) in all criterial test items (back-transformed from the logits in the GLMM) by retrievability and review condition (testing vs. restudy).

The model estimates for the effects of testing with short-answer questions can be found in Table 2 (left columns). This model revealed a positive effect for testing (β = 0.44, *SE* = 0.24, *p* = 0.033, one-tailed). However, the interaction of testing vs. restudying with the predictor comparing low to high retrievability was significant (β = −0.60, *SE* = 0.28, *p* = 0.016, one-tailed). Likewise, the interaction with the predictor comparing medium to high retrievability was significant (β = −0.66, *SE* = 0.35, *p* = 0.030, one-tailed). Planned contrasts revealed a testing effect only for items with high retrievability (*z* = 1.85, *p* = 0.032, one-tailed) but not for items with medium (*z* = −0.74, *p* = 0.771, one-tailed) or low retrievability (*z* = −0.66, *p* = 0.746, one-tailed) (Figure 2).

**Table 2:** Parameter estimates for the models estimating the effect of testing with short-answer questions and multiple-choice questions, time of test, and retrievability on short-answer questions and multiple-choice questions on learning performance in the criterial tests.

	Short-answer questions				Multiple-choice questions			
Parameter	*β*	*SE*	*z*	*p*	*β*	*SE*	*z*	*p*
Intercept	-0.34	0.25	−1.36	0.173	0.07	0.29	0.25	0.803
Testing	0.44	0.24	1.84	0.033^a^	−0.42	0.24	−1.76	0.078
Criterial test 2	0.80	0.27	2.96	0.003	0.74	0.29	2.55	0.011
Criterial test 3	0.11	0.38	0.30	0.768	0.38	0.42	0.91	0.361
Testing × Criterial test 2	−0.14	0.22	−0.63	0.531	0.12	0.22	0.55	0.583
Testing × Criterial test 3	−0.23	0.32	−0.73	0.468	−0.11	0.34	−0.33	0.739
Low retrievability	0.03	0.25	0.10	0.917	−0.31	0.25	−1.23	0.219
Medium retrievability	0.09	0.23	0.40	0.692	−0.35	0.27	−1.33	0.184
Testing × Low retrievability	−0.60	0.28	−2.14	0.016^a^	0.17	0.27	0.62	0.534
Testing × Medium retrievability	−0.66	0.35	−1.88	0.030^a^	0.060	0.37	0.16	0.872
*N*_Participants_	92				91			
*N*_Items_	77				77			

*Testing (dummy-coded: testing = 1, restudy = 0). Criterial test 2 (dummy-coded: Criterial test 2 = 1, Criterial test 1 = 0). Criterial test 3 (dummy-coded: Criterial test 3 = 1, Criterial test 1 = 0). Low retrievability (dummy-coded: low retrievability = 1, high retrievability = 0). Medium retrievability (dummy-coded: medium retrievability = 1, high retrievability = 0). ^a^p-values refer to one-tailed tests for β > 0. Other p-values refer to two-tailed tests.*

The interactions with time of tests were not significant, suggesting that the testing effect obtained for short-answer questions was independent of the time of test. However, there was a main effect of the predictor comparing Criterial Test 2 to Criterial Test 1. The probability of giving a correct response was higher at Criterial Test 2 (*P* = 0.61, *SE* = 0.04) compared to Criterial Test 1 (*P* = 0.43, *SE* = 0.05).

### Effects of Testing With Multiple-Choice Questions

The model estimates for the effects of testing with multiple-choice questions can be found in Table 2 (right columns). No effect of testing vs. restudying emerged. None of the interaction effects of testing with time of test or retrievability were significant. Again, there was a main effect of the predictor comparing Criterial Test 2 to Criterial Test 1. The probability of correct responses was higher at Criterial Test 2 (*P* = 0.62, *SE* = 0.05) compared to Criterial Test 1 (*P* = 0.42, *SE* = 0.05).

In sum, the results indicated no testing effect for multiple-choice questions.

## Discussion

The present study investigated the testing effect in a university education setting by implementing a minimal intervention in an existing university course. In contrast to many previous studies with a similar aim, we took care to avoid confounding factors and based our study on an experimental design. The main finding was a testing effect for practice tests based on short-answer questions, provided that participants in the testing condition were able to retrieve this content. No evidence was found for a testing effect for practice tests based on multiple-choice questions.

Our study method shares many features with lab experiments investigating the net testing effect (e.g., Roediger and Karpicke, 2006a, Experiment 1), with the obvious difference being that the setting of the current experiment was in real-world educational context. Although this difference alone could have contributed to the lack of an overall testing effect, two other factors are likely to affect the testing effect in laboratory and educational contexts. Most research uses a repetition of the entire learning content in the restudy condition, but exact repetitions are difficult to implement in real-world educational contexts because of time constraints, that is, usually only selected information is restudied. Participants in our study studied summaries of important aspects of the lecture. In this regard, Kornell et al. (2012) argued that restudying the material in the same way might overestimate the testing effect, but they also provided evidence that testing might be superior to restudying non-exact repetition of study material.

The testing effect for practice tests based on short answer questions depended on retrievability of the initially learned content. A testing effect occurred only for questions with a high retrievability, that is, mean retrievability rates between 46 and 81%. This finding is in line with previous findings from laboratory experiments (Rowland, 2014) and with the bifurcation model (Halamish and Bjork, 2011; Kornell et al., 2011). The bifurcation model states that the superiority of testing without feedback compared to restudying depends on the amount of successfully retrieved items in the testing condition. Support for the bifurcation model comes from the meta-analysis by Rowland (2014) that revealed no testing effect for laboratory experiments with no corrective feedback and retrievability rates of less than or equal to 50%. Our findings can thus be regarded as additional support of the bifurcation model in educational contexts. These findings also extend the existing research, because the testing effect, although implemented through a minimalistic intervention, was stable over a period of at least 23 weeks.

In line with findings from lab experiments investigating the net testing effect, a testing effect emerged for short-answer questions after a single presentation of these questions. Lab experiments investigating repeated testing without feedback also revealed a net testing effect (Roediger and Karpicke, 2006a, Experiment 2; Wirebring et al., 2015). Repeated short-answer testing might be even more potent in an educational setting than short-answer testing on a single occasion. Future studies should compare these two ways to implement short-answer testing in educational settings.

In contrast to testing based on short-answer questions, no testing effect emerged for practice tests based on multiple-choice questions. This pattern of effects is in line with current theories of the testing effect that emphasize the role of cognitive effort during retrieval (Bjork, 1994; Pyc and Rawson, 2009). Questions that prompt effortful retrieval are likely to elicit stronger testing effects. The multiple-choice questions used in the present study were relatively easy (compared to the short-answer questions). Two-thirds of the items were solved correctly in most of the cases, suggesting that participants spent relatively little effort in retrieving the relevant information from long-term memory. Moreover, multiple-choice questions may have a negative effect on learning retention because of the presence of distractors (lures). Roediger and Marsh (2005) have shown that multiple-choice testing may lead participants to answer later criterial tests with false information. Further research suggests that this impact can be lessened by corrective feedback (Marsh et al., 2012). In the present study, no corrective feedback was given, implying that the distracting information could have influenced the performance on the criterial tests, counteracting the testing effect.

The experimental design in a field study is a strength of the present study, but the method also presents some limitations. Compared to laboratory experiments, external influences potentially play a much greater role in a field setting. For the present study, the extent that other factors (e.g., metamemorial, metacognitive, or motivational factors) influenced learning behavior during lectures and review conditions, when taking the criterial tests, or in the days and weeks between the lectures and the criterial tests is unknown. For example, the performance in the criterial tests increased steeply from the first to the second criterial test, which is likely caused by participants’ increased study activities in preparation for the upcoming exam. Participation in the study in each of the lectures was voluntary, which might have caused selection effects. However, it must be noted that these selection effects likely affected all experimental conditions to the same extent, because participants were unaware of the review condition that they would be assigned to when they made their decision to participate.

Another limitation that our study shares with other studies on the testing effect is the potential confound of test properties for the practice and criterial tests. For example, multiple-choice questions not eliciting a testing effect might be due to the low demand on retrieval effort involved in answering multiple-choice questions (e.g., Nguyen and McDaniel, 2015). Thus, drawing conclusions that multiple-choice questions are generally unsuitable for eliciting a testing effect would be premature.

To conclude, this research contributes to the literature by demonstrating a testing effect for practice tests with short-answer questions in the real-world educational context of a university lecture. Previous research has examined the testing effect, normally combined with additional features or based on quasi-experimental designs, which has hindered interpretation of the testing effect reported in these studies. In contrast, the present study provides clear evidence for the claim that answering short-answer questions only once and without feedback, compared to restudying key points of the lecture, benefits retention of learning content even beyond the final exam. However, one important condition is that the difficulty of these questions must be at a level such that students are able to answer most of these questions correctly. To use the testing effect to foster learning, educational practitioners should identify the most important topics of their lecture, teach these thoroughly, and use short-answer testing to solidify the knowledge about these topics. Finally, presenting students with multiple-choice questions might be ineffective, compared to restudying key points of the lecture. Given these findings, we advise practitioners to use short-answer testing rather than multiple-choice testing to foster learning in university lectures.

## Data Availability

After publication, the data files underlying the analyses reported in this study will be made publicly available via Open Science Framework [www.osf.io].

## Ethics Statement

For the reported study, no ethics approval was required per the guidelines of the University of Kassel or national guidelines.

## Author Contributions

TR: supervision of the project, design of the research, and revision of the article. SG: design of the research, organization of experiment conduction, data analysis and interpretation, and writing of the article.

## Conflict of Interest Statement

The authors declare that the research was conducted in the absence of any commercial or financial relationships that could be construed as a potential conflict of interest.
